# Mobile Text Messaging to Improve Medication Adherence and Viral Load in a Vulnerable Canadian Population Living With Human Immunodeficiency Virus: A Repeated Measures Study

**DOI:** 10.2196/jmir.6631

**Published:** 2017-06-01

**Authors:** Elizabeth King, Karen Kinvig, Jonathan Steif, Annie Q Qiu, Evelyn J Maan, Arianne YK Albert, Neora Pick, Ariane Alimenti, Mary H Kestler, Deborah M Money, Richard T Lester, Melanie Caroline Margaret Murray

**Affiliations:** ^1^ Department of Medicine University of British Columbia Vancouver, BC Canada; ^2^ Oak Tree Clinic British Columbia Women's Hospital Vancouver, BC Canada; ^3^ Department of Mathematics University of British Columbia Vancouver, BC Canada; ^4^ Women's Health Research Institute British Columbia Women's Hospital Vancouver, BC Canada; ^5^ Division of Infectious Diseases Department of Medicine University of British Columbia Vancouver, BC Canada; ^6^ Department of Pediatrics University of British Columbia Vancouver, BC Canada; ^7^ Department of Obstetrics and Gynecology University of British Columbia Vancouver, BC Canada

**Keywords:** mHealth, HIV, medication adherence, vulnerable, female, viral load, mobile phone, engagement

## Abstract

**Background:**

Combination antiretroviral therapy (cART) as treatment for human immunodeficiency virus (HIV) infection is effective and available, but poor medication adherence limits benefits, particularly in vulnerable populations. In a Kenyan randomized controlled trial, a weekly text-messaging intervention (WelTel) improved cART adherence and HIV viral load (VL). Despite growing evidence for short message service (SMS) text-message interventions in HIV care, there is a paucity of data utilizing these interventions in marginalized or female cohorts.

**Objective:**

This study was undertaken to assess whether the standardized WelTel SMS text-message intervention applied to a vulnerable, predominantly female, population improved cART adherence and VL.

**Methods:**

We conducted a repeated measures study of the WelTel intervention in high-risk HIV-positive persons by measuring change in VL, CD4 count, and self-reported adherence 12 months before and 12 months after the WelTel intervention was introduced. Inclusion criteria included VL ≥200 copies/mL, indication for treatment, and meeting vulnerability criteria. Participants were given a mobile phone with unlimited texting (where required), and weekly check-in text messages were sent for one year from the WelTel computer platform. Clinical data were collected for control and intervention years. Participants were followed by a multidisciplinary team in a clinical setting. Outcomes were assessed using Wilcoxon signed ranks tests for change in CD4 and VL from control year to study end and mixed-effects logistic regressions for change in cART adherence and appointment attendance. A secondary analysis was conducted to assess the effect of response rate on the outcome by modeling final log_10_ VL by number of responses while controlling for mean log_10_ VL in the control year.

**Results:**

Eighty-five participants enrolled in the study, but 5 withdrew (final N=80). Participants were predominantly female (90%, 72/80) with a variety of vulnerabilities. Mean VL decreased from 1098 copies/mL in the control year to 439 copies/mL at study end (P=.004). Adherence to cART significantly improved (OR 1.14, IQR 1.10-1.18; P<.001), whereas appointment attendance decreased slightly with the intervention (OR 0.81, IQR 0.67-0.99; P=.03). A response was received for 46.57% (1753/3764) of messages sent and 9.62% (362/3764) of text messages sent were replied to with a problem. An outcome analysis examining relationship between reply rate and VL did not meet statistical significance (P=.07), but may be worthy of investigating further in a larger study.

**Conclusions:**

WelTel may be an effective tool for improving cART adherence and reducing VLs among high-risk, vulnerable HIV-positive persons.

**Trial Registration:**

Clinicaltrials.gov NCT02603536; https://clinicaltrials.gov/ct2/show/NCT02603536 (Archived by WebCite at http://www.webcitation.org/6qK57zCwv)

## Introduction

In Canada, approximately 75,500 people are living with human immunodeficiency virus (HIV), 16,800 of whom are women [1]. Combination antiretroviral therapy (cART) has led to enormous improvements in health and survival of HIV-positive individuals [2]. Moreover, by decreasing the amount of virus circulating in the plasma (viral load [VL]), cART offers the possibility of treatment as prevention by minimizing viral transmission [3,4]. Despite the well-established benefits of cART, nonadherence to medications, delayed initiation of therapy, and poor follow-up are ongoing limitations of effective HIV management leading to multidrug resistance, progression to acquired immune deficiency syndrome (AIDS), transmission to others, and mortality [2,5-7].

Engagement in longitudinal HIV care is an ongoing challenge, with studies from the United States showing long-term retention in care ranging from 37% to 65% [8-11]. Retention in care is particularly important on a public health scale because those individuals not retained in care have been estimated to account for twice as many HIV transmissions as nondiagnosed individuals [11]. In British Columbia, Canada, there is emerging evidence that cART adherence among women is lagging behind that of men (59% vs 68%, respectively) [12]. A variety of factors have been shown to decrease care retention and cART adherence in women, including intimate partner violence, medication side effects, role as care provider, and gender-specific stigma [13-19]. Other risk factors associated with poor adherence in both men and women include substance abuse, recent incarceration, psychiatric conditions, advanced HIV, and lack of social or housing supports [8,20-25]. Previous peer mentoring and behavioral interventions tested in these populations have failed to show objective improvements in HIV care [26-28]. For an illness that now has beneficial long-term treatment available, effective adherence strategies for difficult-to-engage populations will be of paramount importance from an individual and public health standpoint.

Mobile health (mHealth), the use of mobile phone technology to deliver health care, is an emerging area of disease management that can assist in patient adherence to prolonged chronic treatment regimens and monitoring of care [29-31]. Short message service (SMS) text messaging is particularly appealing as a mHealth intervention because it makes use of standard services offered by cellular providers, requires minimal additional infrastructure, and is user-friendly, ubiquitous, and flexible [29,30,32]. In a landmark randomized controlled trial, Lester et al conducted a mHealth intervention (WelTel Kenya1) whereby providing a bidirectional, weekly text-messaging intervention for one year significantly improved cART adherence and VL suppression compared with controls in an HIV-positive Kenyan population initiating cART [16]. Several other SMS text-message interventions have been developed and implemented in HIV care with promising results; however, WelTel remains the approach validated in the largest setting to date [33-35]. In addition, reviews and a meta-analysis have assessed available evidence to confirm that SMS text-message interventions can improve parameters of HIV care, especially cART adherence [17,36-38]. Despite this emerging evidence, there remains a lack of focus on vulnerable populations less likely to access care and a paucity of data focused on female populations [39-42]. Our goal in this study was to assess whether the standardized WelTel intervention applied to a vulnerable, predominantly female population improved cART adherence and VL.

## Methods

### Study Participants

Participants were enrolled in a repeated measures cohort study between April 2013 and May 2014 with the 12 months prior to initiation of the study used retrospectively as the control year. Participants were recruited from the Oak Tree Clinic in Vancouver, BC, Canada, which is a provincial referral center for women and families living with HIV throughout British Columbia, many of whom face multiple barriers to engagement in care. The clinic offers an interdisciplinary care team consisting of physicians, nurses, pharmacists, social workers, dieticians, and counselors addressing the holistic health needs of women and their families in a single care setting.

**Figure 1:**
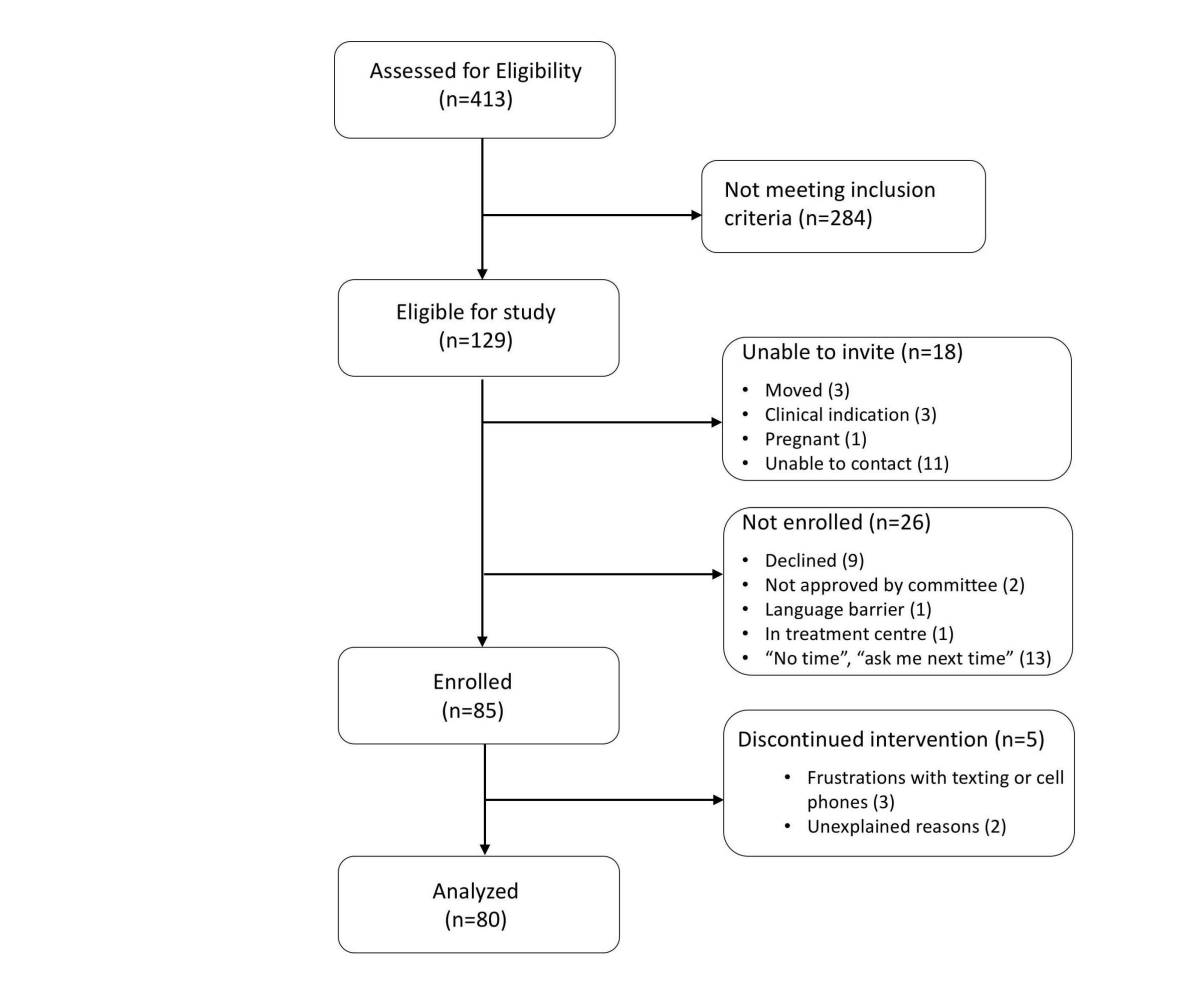
CONSORT flow diagram of trial.

As outlined in Figure 1, 413 individuals were assessed for eligibility, 129 were eligible, and 85 enrolled in the study. Five participants dropped out of the study early on and were not included in the final analysis. HIV-positive patients were eligible for study participation if they met the following inclusion criteria: attendance at the clinic for at least one year prior to study entry, an indication for cART (at time of study development, CD4 <500 cells/mm^3^), detectable VL (≥200 copies/mL) in the year prior to study entry (control year), age 14 years or older, and high risk for disengagement in treatment according to a list of predetermined criteria. High-risk individuals were identified based on care team consensus that at least one of the following vulnerability criteria were present: intimate partner violence, unstable housing, advanced HIV infection/AIDS, mental health illness, cART nonadherence, difficult to contact, poor appointment attendance, active substance use, long distance from care, or recent incarceration. All candidates were reviewed by the multidisciplinary care team that decided on applicable vulnerabilities by manner of consensus. We excluded those who did not meet the preceding criteria or lived in an area with no cell phone service. Those who were unable to communicate by text messaging due to language barriers, illiteracy, or physical disabilities preventing use were also excluded. For those participants aged 14 to 18 years, parental consent for enrollment in the study was not obtained in an effort to protect confidentiality and promote safety in this marginalized cohort.

Prospective participants were introduced to the WelTel intervention during a routine clinical visit and provided written informed consent if interested in the study. Participants were given a basic cell phone with unlimited text-messaging capability if they did not have one. For those patients that did own a phone with a text-messaging plan, no payment incentive was provided. However, if an individual required a phone or phone plan at a later time, they were provided with one. When required, participants received instruction on how to use text messaging for communication. In addition to the intervention, participants continued to receive their regular care through the interdisciplinary team. In British Columbia, cART is fully funded for HIV-positive patients through the Provincial Antiretroviral Drug Treatment program and was prescribed according to published therapeutic guidelines by the BC Centre for Excellence in HIV/AIDS [43]. The study protocol was approved by the University of British Columbia Research Ethics Board (H12-03002) and registered as a clinical trial (NCT02603536).

### Intervention

The SMS text-message intervention was modeled after the WelTelKenya1 study with participant-driven modifications according to a qualitative assessment conducted with patient participants and health care providers at the clinic prior to study start [16,18,44]. The WelTel model was selected because it has been validated in the largest setting to date and contains many features (ie, weekly, short, bidirectional messages) that have been subsequently shown to be effective SMS text-message strategies [16,34]. For one year, the text message “How are you?” was sent to all participants every Monday at noon from the automated WelTel platform through a number not traceable to the clinic. Participants were instructed to respond “OK” or “not OK,” and those responding “not OK” received a follow-up call by a study nurse within approximately 24 hours. If no response was received from the initial SMS text message, a second text message was sent on Wednesday stating, “Haven’t heard from you yet, how are you?” If there was still no response, this was followed by a call on Thursday by study nurses. Participants were instructed that the intervention was not an emergency service and were provided with a written reminder of the current practices for after-hours concerns and emergencies. This open-ended check-in approach was specifically designed to preserve confidentiality and provide a personal connection to clinic staff to triage all problem types. For confidentiality reasons, health care providers did not text information relating to HIV status unless asked explicitly to do so by the participant. Participants who lost their own phone or the study phone were offered a replacement on a one-time basis. Participants were allowed to reinitiate the study at any time simply by providing the team with the new phone number.

### Measuring Outcomes

The primary outcome of the study was change in VL from control year (mean VL over one year prior to study entry) to final VL at study end. Secondary outcomes included change in cART adherence, CD4 count, and appointment attendance following the WelTel intervention. Parameters anticipated to respond after a lag time (VL and CD4) were compared using a mean of all measurements in the control year to final values at the study end, whereas those expected to have rapid response to the intervention (cART adherence and appointment attendance) were assessed by comparing means of control and intervention years in their entirety. To estimate the number of cART doses taken, self-reported adherence was collected at each medical visit with appointments arranged at 1- to 4-month intervals according to health status. At each visit, the patient was asked to estimate the number of ingested doses and missed doses since the past visit. Self-reported adherence was then compared to pharmacy refill records, always favoring the lowest estimate of doses taken. Finally, adherence was then calculated as the percentage of estimated doses taken from the total number of doses prescribed in the year. Appointment attendance data was gathered from the electronic booking system for the clinic.

Data were collected for one year prior to texting start date and for the duration of the study such that each participant served as their own control in the intervention. Participant self-reported ethnicity and vulnerability data were collected at time of enrollment. Chart review was used to collect the following: age, housing status, substance use, geographic location, CD4 count and percentage, VL, cART regimen (including dates of initiation or discontinuation), emergency department visits, hospital admissions, self-reported medication adherence, pharmacy refill data, and clinic appointment attendance. All text-messaging responses, follow-up calls, and team member referrals were tracked and nature of the problem noted.

### Statistical Analysis

A sample size of 100 was initially targeted based on pilot study values of the primary outcome (VL) to allow for statistical assessment with a power of 80% and significance level of .05. To assess changes in VL and CD4, we computed the geometric mean (log_10_-transformed for VL) from raw data of all laboratory results from the control year for each participant. These means were then compared to the final VL or CD4 from the intervention year using nonparametric Wilcoxon signed ranks tests for paired data. Odds ratios for cART adherence and clinic attendance were analyzed based on binomial “yes/no” responses for each event with several events for each participant and then calculated by logistic regression. For each participant, the number of possible appointments and doses was tallied within each year, and the proportion of those that were attended/taken was compared between years using mixed-effect logistic regressions to control for paired measurements for each participant. Given the bidirectional nature of the text-messaging intervention, participants and care providers were not blinded throughout. In addition, outcomes were assessed according to the pre/post intervention study design rather than blinded.

To facilitate a comparison between those patients regularly using mHealth technology versus those not actively using it, we analyzed variation in the number of responses to the text messages. All participants should have received 52 messages making the number of responses a useful proxy of engagement. We used bivariate negative binomial regression to analyze the number of responses by age, gender, ethnicity, income, long-distance telephone, distance traveled to clinic (≤50km, >50km), any substance use (not including smoking), and mean log_10_ VL during control year. We estimated the effect of response rate on the outcome by modeling final log_10_ VL by number of responses while controlling for mean log_10_ VL in the control year using a general linear model.

## Results

### Cohort Characteristics

Between April 2013 and May 2014, 85 participants were enrolled in the study. Although an initial target of 100 patients was selected based on statistical analysis, there were limitations in eligible clinic patients for enrollment. As such, study size was reanalyzed and determined to have sufficient power with a modified goal of 85 participants. Five participants withdrew from the study. Baseline demographics of the remaining participants are outlined in Table 1. The population was predominantly female (90%, 72/80) with a median age of 38 (IQR 29-47, range 15-61) years. Six (8%, 6/80) participants were younger than 18 years and received pediatric care at the clinic. The majority of the cohort had multiple vulnerabilities (76%, 62/80) of which cART nonadherence (54%, 44/80), mental health illness (48%, 39/80), active substance use (28%, 23/80), and unstable housing (28%,14/80) were the most common (Table 1). Almost half of participants did not own a cell phone at the time of enrollment (46%, 37/80). Geographic distance varied widely; however, the majority of participants lived within 50 km of the clinic. Final recorded VL measurements were taken a median of 314 days (IQR 269-348) after initiation of text messaging.

### Intervention Efficacy

**Figure 2:**
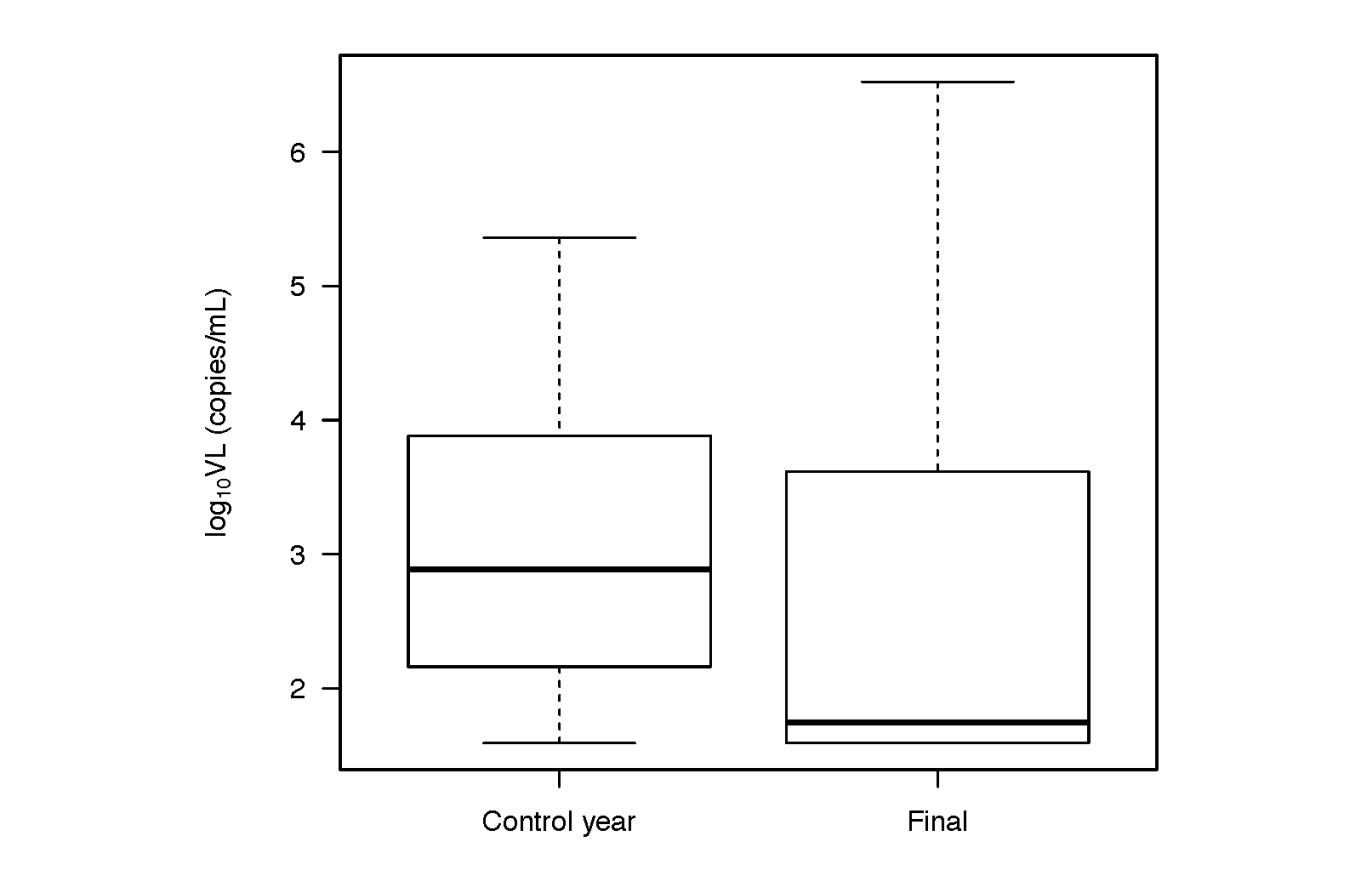
Boxplot of mean log_10_ VL in the control year compared to the log_10_ VL at study end. The black bars indicate the medians; boxes indicate the interquartile range and the whiskers extend to 1.5× the interquartile range. There was a significant decrease in log_10_ VL after the intervention (P=.004).

**Figure 3:**
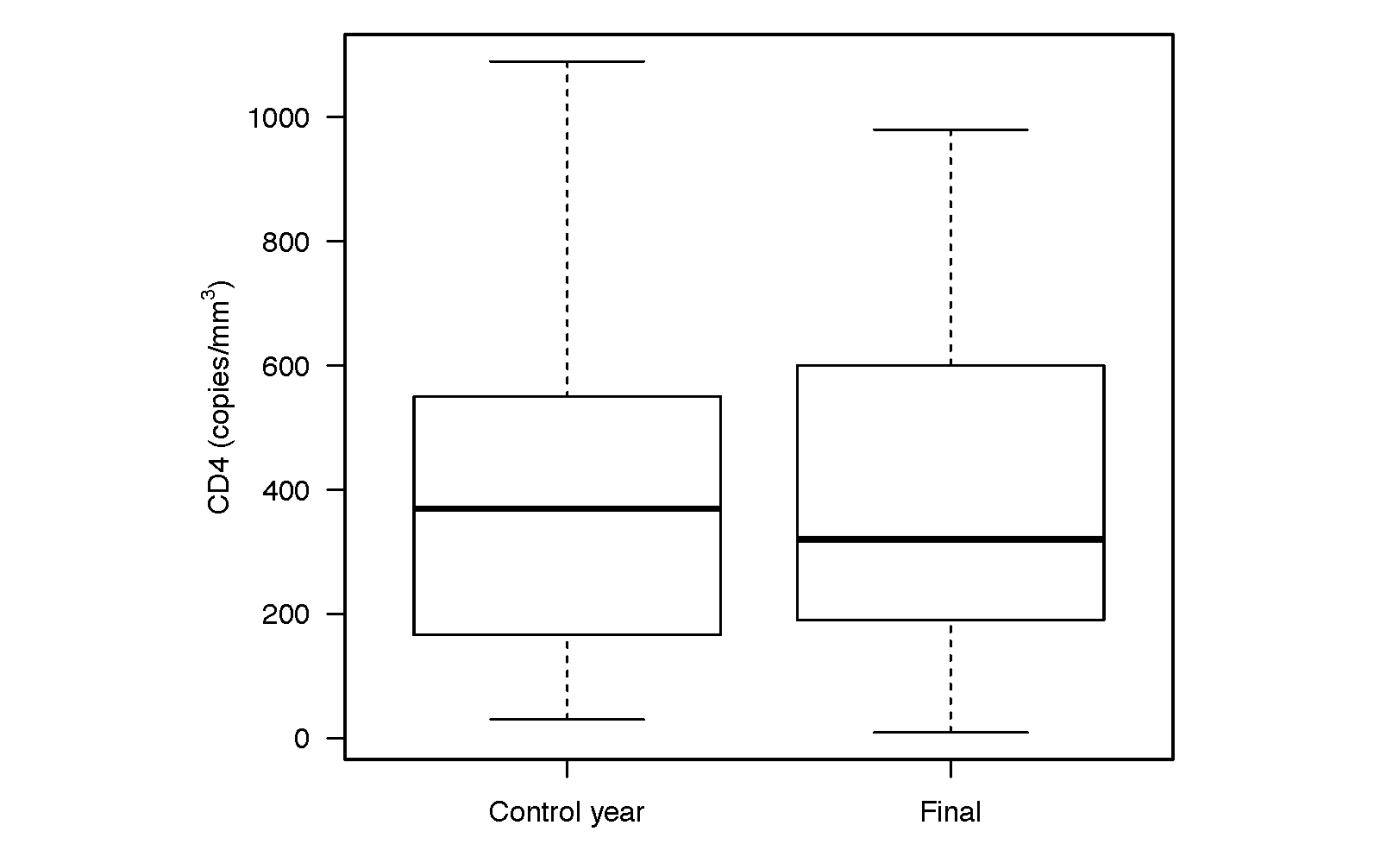
Boxplot of the mean CD4 in the control year compared to the mean CD4 at the study end. The black bars indicate the medians; boxes indicate the interquartile range and the whiskers extend to 1.5× the interquartile range. There was no significant change in CD4 after study intervention.

Viral load, the primary outcome, significantly decreased from control year to study end (*P*=.004; Table 2 and Figure 2). Overall, geometric mean VL decreased from mean 1098 (95% CI 647-1866) copies/mL in the control year to mean 439 (95% CI 217-888) copies/mL at intervention end (log_10_ VL 3.04-2.64; Cohen’s *d*=0.33, 95% CI 0.02-0.65), with 38 of 80 participants having undetectable VL at study end. In contrast, CD4 did not significantly change postintervention (Table 2 and Figure 3). Of the secondary outcomes, cART adherence significantly improved from control year to intervention year with the odds of adherence increasing by 14% during the WelTel intervention (OR 1.14, IQR 1.10-1.18, *P*<.001; Table 2). Finally, there was a 19% reduction in the odds of attending clinic appointments in the intervention year (OR 0.81, IQR 0.67-0.99, *P*=.03). Other variables, including CD4 count, substance use, cART regimen, and hospital admissions, did not significantly change postintervention (Table 2).

**Table 1:** Baseline demographics of a high-risk Canadian HIV-positive cohort (N=80).

Demographics			Participants
**Gender, n (%)**			
	Female		72 (90)
	Male		6 (8)
	Transgender		2 (3)
Age (years), median (range)			38 (15-61)
**Ethnicity, n (%)**			
	Caucasian		30 (38)
	First Nations		27 (34)
	African Canadian		18 (22)
	South Asian		5 (6)
**Income source, n (%)**			
	Disability		57 (71)
	Welfare		6 (8)
	Employed		4 (5)
	Other		13 (16)
**Housing status, n (%)**			
	Stable housing		57 (71)
	**Unstable housing**		23 (29)
		Unsheltered	1 (1)
		Emergency sheltered	9 (11)
		Provisionally sheltered	13 (16)
**Vulnerability,^a^n (%)**			
	Multiple (≥2) vulnerabilities		62 (76)
	Intimate partner violence		4 (5)
	Unstable housing		23 (28)
	Advanced HIV^b^		22 (27)
	Mental health illness		39 (48)
	cART nonadherence		44 (54)
	Difficult to contact		12 (15)
	Poor appointment attendance		22 (27)
	Substance use		22 (28)
	Long distance from care		6 (7)
	Recent incarceration		4 (5)
**Cell phone ownership, n (%)**			
	Yes		43 (54)
	No		37 (46)
Geographic distance (km),^c^ median (IQR, max)			29.6 (7.75-44.9, 1500)

^a^ Vulnerabilities were expressed as percentage of population. Because multiple vulnerabilities were allowed for each individual, combined percentages will exceed 100%.

^b^ Advanced HIV was defined by CD4 count ≤200 cells/mm^3^.

^c^ Geographic distance was calculated as the distance (in km) from the Oak Tree Clinic.

**Table 2:** Outcomes and health measures before and after WelTel intervention for a vulnerable cohort with HIV (N=80).

Outcomes and health measures			Control year	Intervention year	*P*
**Primary outcome**					
	Geometric mean VL (copies/mL), mean (95% CI)		1098 (647-1866)	439 (217-888)	.004
**Secondary outcomes**					
	CD4 (cells/mm^3^),^a^ median (IQR)		370 (166-550)	320 (190-600)	.24
	**Attendance (%), mean (95% CI)**				
		Clinic appointments	52 (48-55)	47 (43-50)	.03
		All appointments	51 (48-55)	48 (45-51)	.12
		cART adherence	60.3 (59.8-60.8)	62.2 (61.6-62.7)	<.001
**Other health measures**					
	Substance use, n (%)				
	Heroin		9 (11)	6 (8)	.45
	Crack/cocaine/crystal meth		15 (19)	14 (19)	>.99
	Polysubstance use^b^		13 (16)	13 (17)	>.99
	Methadone		17 (21)	17 (21)	.68
	**cART regimen,^c^n (%)**				
		Protease inhibitor-based	49 (61)	39 (48)	.29
		Nonnucleoside reverse transcriptase inhibitor-based	10 (12)	7 (9)	.47
		Integrase-based	10 (12)	17 (21)	.18
		Combination	7 (9)	11 (14)	.45
		None	4 (5)	6 (7)	.53
	**Hospital stays^d^**				
		Total visits to ED for cohort, n, individual median (range)	62, 0 (0-11)	77, 0 (0-17)	.30
		Total admissions to hospital, n, individual median (range)	20, 0 (0-2)	28, 0 (0-5)	.23
		Days of hospital stay/admission individual median (IQR)	11.7 (5-11.5)	9.6 (1.8-15.3)	.63

^a^ Viral load and CD4 counts for intervention year were calculated based on study end data: geometric mean (95% CI) for VL and median (IQR) for CD4.

^b^ Polysubstance use was defined as use of more than one of the following substances: alcohol, heroin, crack/cocaine, and methamphetamine.

^c^ The reported cART regimen is the treatment combination used for the majority of study days in the control and intervention periods.

^d^ Visits to the emergency department (ED) and hospital admissions were compared using Wilcoxon rank sum tests.

### Intervention Responses

**Figure 4:**
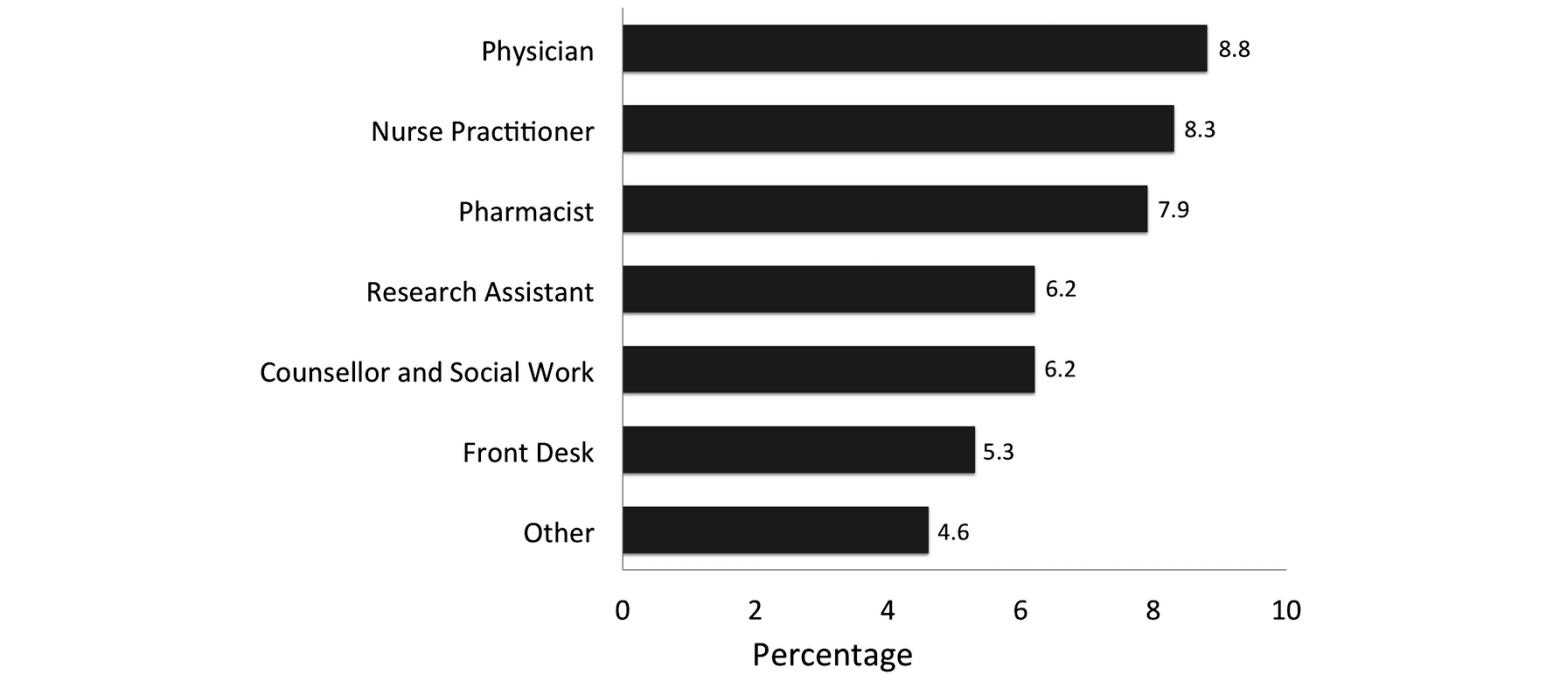
Rate of health care provider involvement for all “problems” identified by SMS text message after assessment by study nurse.

**Figure 5:**
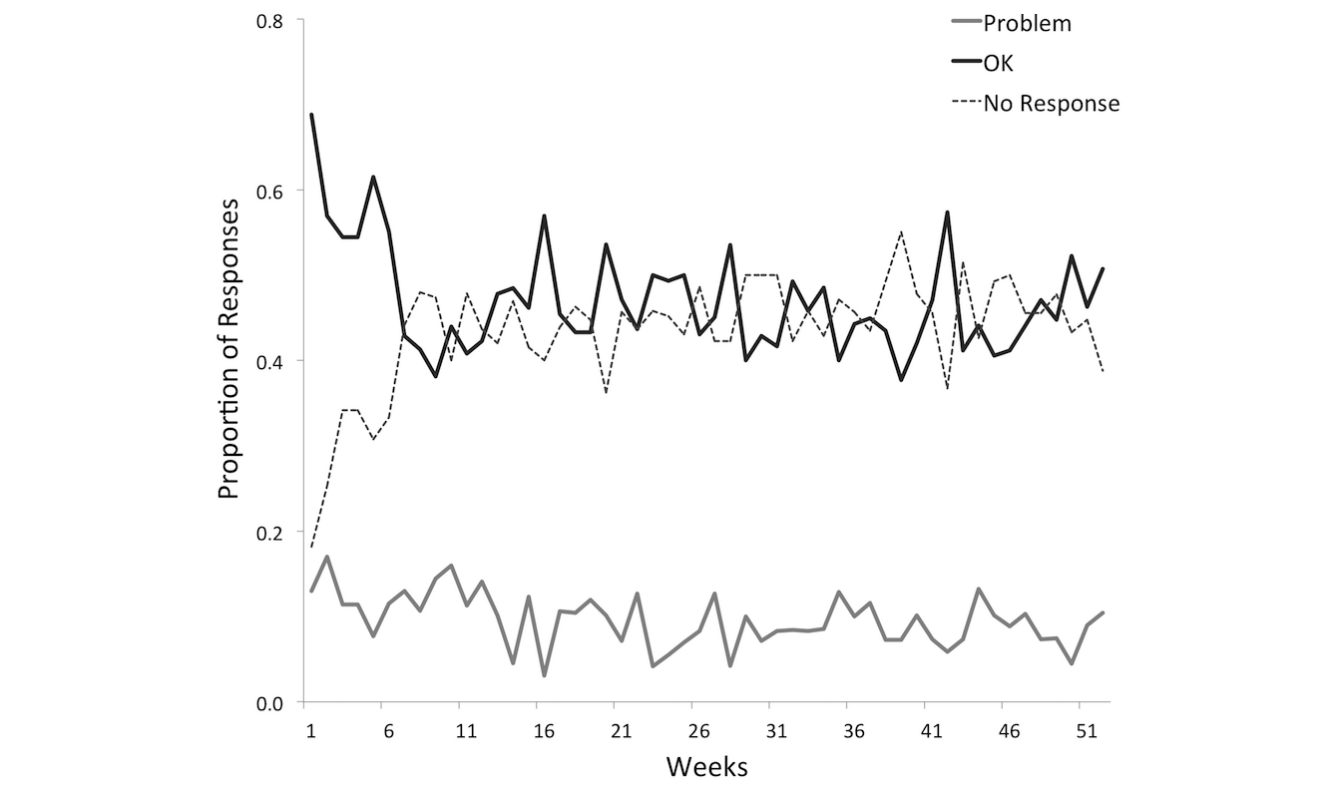
Proportion of weekly SMS text-message response types throughout the intervention.

Of the 3674 text messages sent to the 80 remaining participants over the study period, 46.57% (1753/3764) of messages sent resulted in an initial “OK” reply, whereas 9.85% (362/3674) resulted in a reply of a “problem” and 42.43% (1559/3674) returned no reply. Although 362 “problem” replies were received in response to the Monday text message, an additional 203 “problem” replies were received later in the week, either as a second “problem” or as a new problem after an initial “OK” response. All “problem” replies were triaged by the study nurse. A total of 267 such problems were then referred to another health care team member as outlined in Figure 4. Physicians, nurse practitioners, and pharmacists were most commonly consulted to address a variety of issues that included medical advice, cART prescription refill orders, medication side effects, and medication pick-ups. Fifty participants were provided with a mobile phone at the beginning of the study, whereas 31 individuals lost a phone and required replacement. Response rates to weekly text messages initially started as high as 68% (54/80), but leveled off by the sixth week to an ongoing rate of approximately 45% (36/80) that remained constant to study end (Figure 5). On average, a fairly constant rate of 10% (8/80) of individuals reported “problems” each week.

Individual response rates varied greatly among participants (0%-98%) with a mean of 50% (26/52 responses). Based on these findings, an exploratory subgroup analysis was undertaken to compare demographic characteristics and VL by the number of replies to the text messages. There was no significant difference in the reply rate by any of the demographic or clinical characteristics explored (Table 3).

**Figure 6:**
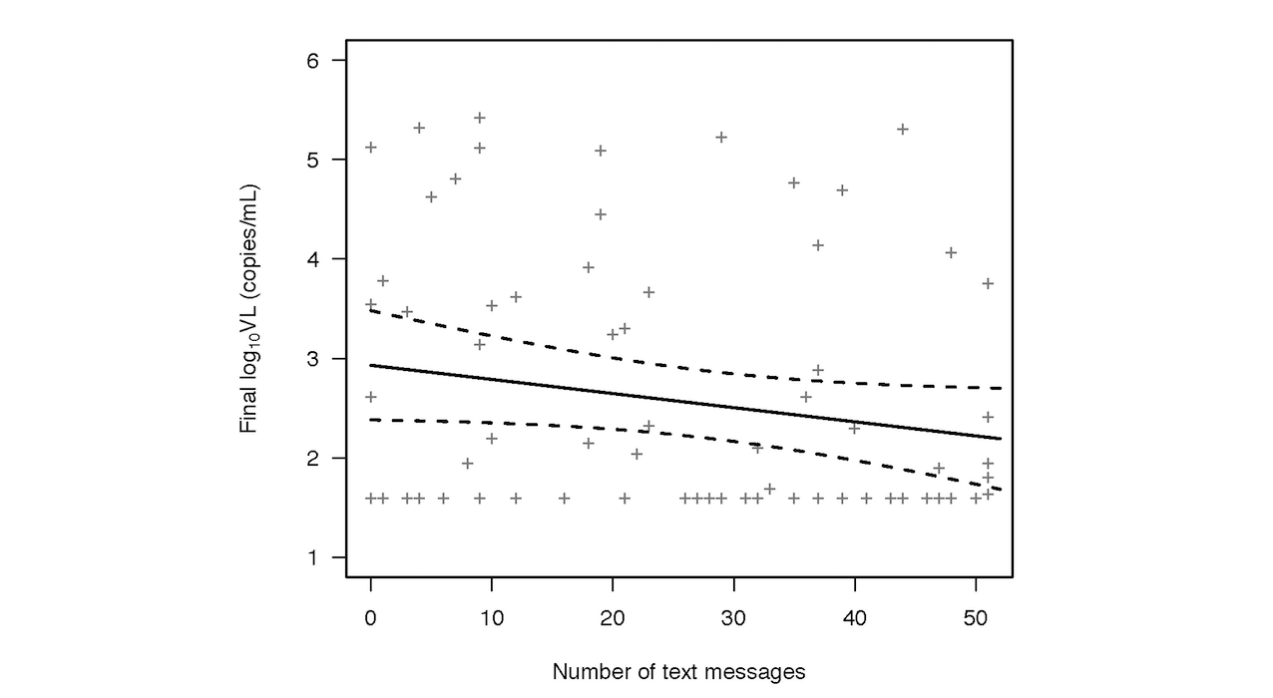
Final log10 VL by number of SMS text-message responses in the intervention year. The solid line indicates predictions from the regression for median control year log10 VL (median 2.88). The dashed lines indicate the 95% CI around the regression estimate.

An outcome analysis of final log_10_ VL by number of replies revealed a trend for a relationship between decreasing VL by increasing reply rate controlling for mean log_10_ VL in the control year (*P*=.07; Figure 6), suggesting that the magnitude of the change in VL during the intervention year was larger for those who were more engaged. The model estimates that for every increase in one reply, the geometric mean VL is reduced by approximately 3% (95% CI 0%-7%).

**Table 3:** Subgroup analysis by number of WelTel text message replies during the study year.

Outcome Variable		n	Incident rate ratio (95% CI)	*P*
**Gender^a^**				
	Male	6	Reference	
	Female	72	1.00 (0.45-1.94)	.99
Age (years)		80	1.00 (0.99-1.02)	.91
**Ethnicity**				
	Caucasian	30	Reference	
	First Nations	27	1.32 (0.84-2.07)	
	African Canadian	18	1.28 (0.78-2.14)	
	South Asian	5	1.50 (0.71-3.68)	.56
**Income source**				
	Disability	57	Reference	
	Other	23	1.18 (0.78-1.81)	.44
**Long-distance call**				
	Yes	15	Reference	
	No	64	0.82 (0.49-1.31)	.42
**Geographic distance**				
	≤50 km	58	Reference	
	>50 km	20	0.85 (0.56-1.32)	.47
**Substance use**				
	None	28	Reference	
	Alcohol or illicit drug use	47	0.75 (0.50-1.11)	.15
Mean control year log_10_ VL		80	0.94 (0.78-1.14)	.54

^a^ The two transgender participants were excluded from this comparison.

## Discussion

This study showed that a cohort of difficult-to-engage, vulnerable individuals living with HIV showed evidence of improved measures of HIV care (VL, cART adherence) when exposed to a weekly bidirectional text-messaging intervention. The study is unique in that participants were provided with mobile phones, allowing for increased engagement of marginalized populations that would otherwise be difficult to access. This intervention could potentially bring substantial health benefits to vulnerable populations in an era in which HIV care is largely limited by medication adherence.

The population demographic was a marginalized, predominantly female cohort with markers of poor HIV engagement. Participants came from a diversity of backgrounds and exhibited the wide range of vulnerabilities and barriers to care that could be encountered in any typical North American urban setting (Table 1; [24,40,45-48]). Vulnerabilities common in our population, including mental illness (48%), active substance use (28%), and unstable housing (17%), have been previously associated with poor cART adherence [24,45-50]. Cell phone ownership in this cohort was lower than average among Canadians (54% vs 82%), further confirming the inaccessible nature of this vulnerable study group [51,52]. By including a majority of women, we propose an adherence strategy applicable for the growing proportion of women living with HIV, a subpopulation that has been minimally studied and has characteristically poor cART adherence [13-15].

Our study demonstrated that effective introduction of the WelTel intervention decreased VL within our cohort. VL in our population was initially 1098 copies/mL, more than double the estimated mean of less than 500 copies/mL in British Columbia [53]. This dropped significantly following introduction of the WelTel intervention to a level that was on par with the provincial average by study end (439 copies/mL, *P=*.004; Table 2). Effective cART therapy is known to reduce viral replication [2]. Therefore, the observed VL reduction is likely a consequence of increased cART adherence (OR 1.14, IQR 1.10-1.18, *P*<.001) and minimizing treatment interruption during the WelTel intervention; we expect this relates to prompt recognition of problems and close connection with care. Due to the nature of studying a high-risk group, there is a risk of exaggerating treatment effects due to anticipated regression to the mean. However, additional analysis suggested that those who regularly used the service (ie, repliers) trended toward greater benefit to VL (Figure 4; *P*=.07). This argues that the improved parameters of HIV care may be due to exposure to the WelTel intervention rather than regression to the mean.

WelTel likely mitigated common barriers to cART adherence by (1) serving as a user-friendly, patient-directed connection tool with the care team, (2) connecting patients in an ongoing fashion with a support network to reduce the effects of isolation and stigma, and (3) aiding health care providers in identifying and resolving problems (both medical and related to the social determinants of health) as they arose, thereby preventing crises. Although the odds of adhering to cART was significantly improved during the intervention (OR 1.14, IQR 1.10-1.18, *P*<.001), the rate of adherence showed only modest improvement (60.3% vs 62.2%, Table 2). We expect that this difference appears small for a variety of reasons, including inaccuracies in self-reported adherence, failure to report treatment interruption, and a large variance in adherence rates. The strategy of early recognition and mitigation of problems has been shown to prevent treatment interruption in other settings as well, such as directly observed treatment of tuberculosis [54]. WelTel appears to improve patient-provider relationships by opening a channel of communication through which both medical concerns and those related to the social determinants of health may be addressed [18,44]. We expect that as these relationships improve and so, in turn, will medication adherence and eventually VL. Taken together, these results suggest that WelTel may be an effective patient-oriented tool to support and improve care in vulnerable HIV-positive populations.

The secondary outcome showing decreased clinic appointment attendance during our intervention year was an unexpected finding (Table 2). In other studies, participant fatigue has occurred with frequent or lengthy messaging interventions [34]. However, our observed consistency in texting response rates suggests that user fatigue does not explain our lower clinic attendance (Figure 3). Instead, we postulate that WelTel addressed common problems that would otherwise be resolved at clinic appointments to the point where participants had a decreased need for clinical appointments [18,29]. Despite having lower clinic attendance after the WelTel intervention, several other markers of health status, such as VL, emergency visits, and hospitalization rates, were the same or improved with the text-messaging intervention (Table 2). A previous hypertension study found that using two-way SMS text-message feedback and telephone consultation as a guide to plan ambulatory care management allowed for improved blood pressure management [55]. This raises the possibility that an effective two-way text-message intervention could be used to help guide HIV ambulatory follow-up frequency, in some cases safely decreasing appointment frequency. Finally, the text-messaging platform could be used to send reminders for upcoming appointments, a technique that has successfully improved clinic attendance in similar studies [34,39,56].

The text-messaging response rate was consistent throughout the study without any tapering trend (Figure 3). Previous studies involving patient participant feedback examined the optimal length and frequency of text messaging and found the best approach to maximize responses was a short weekly SMS text message, as opposed to daily texts or longer messaging interventions [33,34,39]. Our study supports this messaging strategy by showing consistent response rates after a full year of weekly text messages. The consistency in response rates that we observed also suggests that participants continue to make use of the intervention after one year, highlighting its feasibility in promoting long-term patient engagement. Previous studies have shown that mHealth interventions continue to have effect up to 18 months after introduction, but the optimal duration beyond this has not yet been studied nor have we seen whether there is a lasting effect if the intervention is withdrawn [16]. As WelTel moves toward broadening its application, future studies investigating the optimal duration of text-messaging interventions and the durability of its effect would help to guide implementation of this tool. Finally, through our analysis of participant engagement, we observed that some of those participants most poised to benefit from the intervention (ie, less adherent with cART) had poor uptake of the intervention in general. This may be because certain characteristics that result in poor adherence in HIV care also contribute to poor engagement with our intervention. Moving forward, it would be of interest to further delineate participant characteristics to anticipate who may maximally benefit from the intervention and for whom the resources will not be effectively allocated.

This study is a repeated measures study rather than a randomized controlled trial; therefore, we are only able to infer association not causation. WelTel had strong evidence of benefit in previous studies and, as such, our study was designed as a cohort study to offer maximal resources to all individuals in a high-risk population rather than random allocation [16-18]. Our study is limited by its sample size, predominantly female composition, and high-risk population studied, and it is therefore not generalizable to all populations. Medication adherence, one of our secondary outcomes, is a notoriously difficult parameter to study and patient self-reports are susceptible to recall bias and reporting inaccuracies. To mitigate this, we included pharmacy refill data in our assessment of number of doses taken, with the lower value being recorded as the correct one. Despite this, our method may still overestimate the number of pills actually ingested. Another limitation was that a large proportion of our cohort (47%) received a cell phone from us during the study, and we cannot control for all effects of cell phone ownership alone. Owning a mobile phone may impact several social determinants of health by impacting relationships, mobility, mental health, and self-perception [57,58]. Changes in these parameters may have gone on to positively influence outcomes associated with HIV care. Finally, multiple factors contribute to a patient’s engagement in care, decision to initiate cART, and readiness to adhere to medication. Complex issues such as mental health and substance use cannot be solved by text messaging alone. However, it is our hope that this technology may act as a facilitator to establish a meaningful connection to care so that our most vulnerable patients may be better able to utilize and engage in the services available to them.

This study found that a simple and easily implemented mHealth intervention may significantly improve HIV care in vulnerable populations, a finding that holds great potential for real world application. Moving forward, further identification of subgroups poised to benefit most from WelTel will be important, particularly if the intervention includes the provision of a cell phone and phone plan. Finally, a cost analysis would shed light on the feasibility of introducing WelTel in a larger scale to targeted populations. We anticipate that the cost of the intervention would be mitigated by the cost-savings benefit of reduced HIV transmission and decreased use of health care resources associated with effective HIV care [2,59]. Taken as a whole, this study is pivotal in proposing an adherence strategy for at-risk HIV populations that we hope can be applied in a broad clinical context.

## Appendix

Multimedia Appendix 1 Consort E-Health Form.

## Abbreviations

AIDS: acquired immune deficiency syndrome
cART: combination antiretroviral therapy
HIV: human immunodeficiency virus
SMS: short message service
VL: viral load
